# Biases in Race and Ethnicity Introduced by Filtering Electronic Health Records for “Complete Data”: Observational Clinical Data Analysis

**DOI:** 10.2196/67591

**Published:** 2025-03-27

**Authors:** Jose Miguel Acitores Cortina, Yasaman Fatapour, Kathleen LaRow Brown, Undina Gisladottir, Michael Zietz, Oliver John Bear Don't Walk IV, Danner Peter, Jacob S Berkowitz, Nadine A Friedrich, Sophia Kivelson, Aditi Kuchi, Hongyu Liu, Apoorva Srinivasan, Kevin K Tsang, Nicholas P Tatonetti

**Affiliations:** 1 Department of Computational Biomedicine, Cedars-Sinai Medical Center, 700 North San Vicente Boulevard, Pacific Design Center Suite G540, Los Angeles, CA, 90069, United States, 1 424 315 1031; 2 Cedars-Sinai Cancer, Cedars-Sinai Medical Center, Los Angeles, CA, United States; 3 Department of Systems Biology, Columbia University, New York, NY, United States; 4 Department of Biomedical Informatics, Columbia University, New York, NY, United States; 5 Department of Biomedical Informatics and Medical Education, University of Washington, Seattle, WA, United States

**Keywords:** health disparities, data quality, observational research, electronic health records, racial and ethnic biases

## Abstract

**Background:**

Integrated clinical databases from national biobanks have advanced the capacity for disease research. Data quality and completeness filters are used when building clinical cohorts to address limitations of data missingness. However, these filters may unintentionally introduce systemic biases when they are correlated with race and ethnicity.

**Objective:**

In this study, we examined the race and ethnicity biases introduced by applying common filters to 4 clinical records databases. Specifically, we evaluated whether these filters introduce biases that disproportionately exclude minoritized groups.

**Methods:**

We applied 19 commonly used data filters to electronic health record datasets from 4 geographically varied locations comprising close to 12 million patients to understand how using these filters introduces sample bias along racial and ethnic groupings. These filters covered a range of information, including demographics, medication records, visit details, and observation periods. We observed the variation in sample drop-off between self-reported ethnic and racial groups for each site as we applied each filter individually.

**Results:**

Applying the observation period filter substantially reduced data availability across all races and ethnicities in all 4 datasets. However, among those examined, the availability of data in the white group remained consistently higher compared to other racial groups after applying each filter. Conversely, the Black or African American group was the most impacted by each filter on these 3 datasets: Cedars-Sinai dataset, UK Biobank, and Columbia University dataset. Among the 4 distinct datasets, only applying the filters to the All of Us dataset resulted in minimal deviation from the baseline, with most racial and ethnic groups following a similar pattern.

**Conclusions:**

Our findings underscore the importance of using only necessary filters, as they might disproportionally affect data availability of minoritized racial and ethnic populations. Researchers must consider these unintentional biases when performing data-driven research and explore techniques to minimize the impact of these filters, such as probabilistic methods or adjusted cohort selection methods. Additionally, we recommend disclosing sample sizes for racial and ethnic groups both before and after data filters are applied to aid the reader in understanding the generalizability of the results. Future work should focus on exploring the effects of filters on downstream analyses.

## Introduction

The rapid adoption of electronic health records (EHRs) in the past decade has greatly expanded the availability and accessibility of clinical data. This advancement enables health care professionals to harness vast amounts of information, driving medical research, personalized medicine, and overall improvements in health care delivery [1,2]. Additionally, it helps to build big data in health care, providing a foundation for advanced analytics and informed decision-making on a large scale. This supports the training and validation of artificial intelligence methodologies and models, leading to improved diagnostic accuracy, personalized treatment plans, and more efficient health care delivery. Therefore, the collected data significantly influence the results and hypotheses derived from these methods.

To improve diversity in health care, studies must include populations underrepresented in the biomedical, clinical, behavioral, and social sciences, such as individuals from racial and ethnic minority groups, those with disabilities, and people from disadvantaged backgrounds [3]. Fostering diversity is vital for producing more accurate, inclusive research outcomes that reflect the needs of all populations, ultimately leading to more equitable health care and improved patient outcomes [4,5].

However, the usefulness of available clinical data is limited if it does not reliably reflect the diversity of the underlying population. Bias in health care research refers to systematic errors or deviations that lead to inaccurate or skewed results, interpretations, or decisions. It usually occurs when certain factors, whether intentional or unintentional, disproportionately influence the research process, leading to outcomes that do not accurately represent the truth. Thus, scientific progress is delayed, flawed conclusions perpetuated, and disparities in health care outcomes are reinforced [6].

Lack of diversity, which may be due to systemic biases and discrimination against individuals and groups from minoritized populations, can lead to biased research outcomes that exacerbate health disparities [7-10]. This can lead to inaccurate conclusions about treatment or interventions that may not apply equally across different populations. In addition, there are still not enough big clinical longitudinal datasets, which are essential for understanding long-term health trends, progression of diseases over time, and evaluating treatment outcomes.

When conducting observational clinical data analysis, it is often preferable to aim for a dataset that is as complete as possible. However, well-meaning filters that improve completeness may introduce unintended biases in the target population [11].

Data completeness can be defined as the extent to which EHRs or other data sources capture all necessary and relevant information to accurately represent a patient’s medical history, care processes, or outcomes. It includes both the presence and accuracy of essential data elements, such as diagnoses, treatments, laboratory results, and preventive care measures, ensuring a comprehensive and reliable foundation for clinical care, research, and quality assessment [12-14].

In this study, we aim to evaluate the effect of data completeness filters originally used by Weber et al [11] on different datasets and how various filters impact the patient cohort. This work extends the analysis to 4 large datasets, including the All of Us (AoU) dataset, UK Biobank (UKBB), and 2 geographically distinct academic medical centers. Specifically, we focus on identifying race and ethnicity biases introduced by commonly used filters [15].

## Methods

We examined 4 distinct data sources, AoU, UKBB, Columbia University dataset, and Cedars-Sinai dataset comprising approximately 12 million patients. By analyzing the available data and applying each filter, we aimed to investigate the potential biases these filters may introduce.

### All of Us

The AoU study, sponsored by the National Institutes of Health, has enrolled more than 814,000 participants as of June 18, 2024, with 80% of them coming from underrepresented populations [16]. These groups include racial and ethnic minorities, people with disabilities, those in rural or underserved areas, and individuals from lower socioeconomic backgrounds. Figure S1 in Multimedia Appendix 1 (provided by the AoU study) showcases the self-reported races and ethnicities of the participants who have completed the initial steps of the program, providing a diverse representation. The recruitment process spans all regions of the United States.

The AoU workbench encompasses a wealth of information gathered from EHRs, including data from Fitbit devices, survey responses, and socioeconomic factors. Notably, a recent release of data in April 2023 included approximately 245,400 whole genome sequencing records and 312,940 genotyping microarrays, further enhancing the dataset’s depth and potential for analysis.

### UK Biobank

The UKBB is a large-scale, population-based study that aims to improve the prevention, diagnosis, and treatment of various diseases. It involves the collection of extensive health-related data, including genetic information, from over 500,000 participants in the United Kingdom. Participants in the UKBB, recruited at ages 40‐69 years, were registered with the National Health Service. Researchers can download the data through the UKBB’s Data Showcase, which collaborates closely with the European Genome Archive.

### Cedars-Sinai

Cedars-Sinai Medical Center (CSMC) is one of the largest hospitals in California, based in Los Angeles, and serves up to 1 million diverse patients every year across its 40 locations in Southern California. CSMC also serves as a large research center. The studied database comprises over 4 million patients.

### Columbia University

Columbia University Irving Medical Center (CUIMC) is a clinical, research, and educational enterprise located on a campus in Northern Manhattan. They are home to 4 colleges and schools that work on scientific research, education, and patient care. The studied database comprises over 5 million patients.

The self-reported race and ethnicity distributions of each dataset, along with the number of participants in each dataset, are presented in Table 1. In addition to those self-reported categories for race and ethnicity, we defined an all group, which includes every patient in that specific dataset. This group serves as a baseline for comparison within the dataset.

**Table 1.** Self-reported race and ethnicity percentages of each dataset, along with the total number of participants in each dataset.

Dataset	CSMC^a^	CUIMC^b^	All of Us	UKBB^c^
Total patients, n	4,031,307	7,121,848	287,012	502,364
**Race, n (%)**				
American Indian and/or Alaska Native	5695 (0.14)	8275 (0.12)	—^d^	—
Asian	205,978 (5.11)	100,046 (1.40)	8294 (2.89)	11,472 (2.28)
Black or African	373,130 (9.26)	445,623 (6.26)	58,264 (20.30)	3552 (0.71)
Native Hawaiian or Pacific Islanders	7082 (0.18)	6693 (0.09)	344 (0.12)	—
White	1,992,336 (49.42)	1,252,219 (17.58)	154,678 (53.89)	473,353 (94.22)
Mixed	—	—	5000 (1.74)	1731 (0.34)
Other	—	1,037,027 (14.56)	4727 (1.65)	10,394 (2.07)
Unknown	1,447,086 (35.89)	4,271,965 (59.99)	55,705 (19.40)	1931 (0.38)
**Ethnicity, n (%)**				
Hispanic or Latino	344,708 (8.55)	657,288 (9.23)	54,054 (18.83)	—
Non-Hispanic or non-Latino	1,691,775 (41.97)	1,298,181 (18.23)	221,935 (77.32)	—
Unknown	1,994,824 (49.48)	5,166,379 (72.54)	11,023 (3.84)	—

a CSMC: Cedars-Sinai Medical Center.

b CUIMC: Columbia University Irving Medical Center.

c UKBB: UK Biobank.

d Not available.

A straightforward approach to identifying subsets of patients whose data are suitable for research studies is to use heuristic computational filters [11] that exclude patients lacking various types of data in their records. For this study, we evaluated 19 different filters, which can be grouped into 3 categories. The first category is based on patient demographics. This includes filters that check whether the patient has both age and sex recorded (AgeSex), if the patient is alive at the time of the search (Alive), if the patient has a known address or zip code (Address or zip), and a set of age filters. The age filters have been applied to age at the time of any diagnosis, for example, the age filter≥65 selects patients who are 65 years or older than 65 years at the time of any of their recorded diagnoses.

The second category is a record-based filter, which checks whether patients have at least 1 recorded instance of various medical data. These filters are the presence of at least 1 diagnosis, the presence of medication records, and records for outpatient visits.

The last category is the time span or observational period filter, which selects patients who have had multiple interactions with the health care system during a specific period of time. The maximum time window for this category was the 6-year follow-up.

We used 19 filters, originally defined by Weiskopf et al [17] as a metric for evaluating the completeness of EHRs, to build patient cohorts within each dataset. These filters helped identify the types of data available after their application. To maintain consistency across datasets, we applied these filters to the patient populations with EHR data in each dataset. Detailed descriptions of each filter, along with their categories, are presented in Table 2.

**Table 2.** Filters used in our analysis, grouped by category and descriptions of each filter.

Group and filter		Description
**Demographics**		
	Alive	Patient is alive at the time of the query
	AgeSex	Patient has both sex and age recorded
	Age filter ≥18	Patient has a diagnosis at an age included in the filter
	Age filter ≤21	Patient has a diagnosis at an age included in the filter
	Age filter ≤40	Patient has a diagnosis at an age included in the filter
	Age filter ≤65	Patient has a diagnosis at an age included in the filter
	Age filter ≥65	Patient has a diagnosis at an age included in the filter
	Age filter ≤80	Patient has a diagnosis at an age included in the filter
	Address or zip code	Patient has an address or zip code recorded
**Medical interactions**		
	Diagnosis	Patient has at least 1 diagnosis recorded
	Medication	Patient has at least 1 medication prescribed and recorded
	Outpatient visit	Patient has at least 1 outpatient visit recorded
**Observation period**		
	1 week	Patient has a recorded observation period equal or longer than the filter span
	2 weeks	Patient has a recorded observation period equal or longer than the filter span
	1 month	Patient has a recorded observation period equal or longer than the filter span
	6 months	Patient has a recorded observation period equal or longer than the filter span
	1 year	Patient has a recorded observation period equal or longer than the filter span
	2 years	Patient has a recorded observation period equal or longer than the filter span
	6 years	Patient has a recorded observation period equal or longer than the filter span

First, we queried our databases to get a count of all the patients, grouping them by self-reported race and ethnicity. After establishing the initial groups, we applied each filter one at a time to see the effect of that filter on sample availability.

This study aimed to identify the biases of different types of filters that are used by researchers to evaluate data completeness in electronic EHR datasets. Our focus is biases that may be introduced upon applying these filters to races and ethnicities.

We then assessed the statistical significance of the filters’ impact on different racial and ethnic subgroups through binomial testing. Comparing the expected sample to the observed filtered sample. For each group, we calculated the *P* values by comparing the observed proportion relative to the sum of that group and the white baseline group against an expected baseline.


$$Observed\;proportion=\frac{filtered\;group\;n}{filtered\;group\;n+filtered\;White\;n}$$

$$Expected\;proportion=\frac{group\;total\;n}{group\;total\;n+White\;total\;n}$$

Subsequently, we adjusted the *P* values for multiple hypothesis correction using the Bonferroni method.

### Ethical Considerations

The research performed complies with all relevant ethical regulations; the institutional review boards (IRBs) that approved the study protocol are Columbia (IRB AAAL0601) and CSMC (IRB STUDY00003395). Patients were enrolled under a waiver of consent in CSMC and CUIMC. Consent in AoU and UKBB is managed by each platform, respectively. During the study, we only accessed nonprotected health information and total counts, maintaining the confidentiality of every patient.

## Results

### Overview

We applied the filters to each dataset separately to assess their individual effects. Table 3 indicates the percentage of patients remaining after applying each filter for each dataset.

**Table 3.** Percentage of population remaining on each dataset after applying the different filters.

Filter	Cedars-Sinai, n (%)	Columbia, n (%)	All of Us, n (%)	UKBB^a^, n (%)
Alive	3,852,435 (95.56)	6,774,829 (95.12)	283,806 (98.88)	467,333 (93.02)
AgeSex	4,026,072 (99.87)	6,002,422 (84.28)	28,701 (100)	502,364 (100)
Age filter ≥18	1,624,951 (40.30)	4,286,839 (60.19)	253,948 (88.48)	229,960 (45.77)
Age filter ≤21	127,771 (3.16)	1,328,385 (18.65)	21,881 (7.62)	82,953 (16.51)
Age filter ≤40	733,191 (18.18)	2,814,216 (39.51)	103,431 (36.04)	178,503 (35.53)
Age filter ≤65	1,348,000 (33.43)	4,393,121 (61.98)	227,072 (79.12)	225,790 (44.49)
Age filter ≥65	424,790 (10.53)	1,170,388 (16.43)	81,938 (28.55)	122,327 (24.35)
Age filter ≤80	1,584,518 (39.30)	4,992,410 (70.09)	252,875 (88.10)	229,961 (45.77)
Has address or zip	1,829,303 (45.37)	4,024,099 (56.50)	287,007 (99.99)	148,261 (29.51)
Has medications	1,316,735 (32.66)	2,513,715 (35.29)	239,691 (83.51)	368,599 (77.37)
Has diagnoses	1,663,429 (41.26)	5,158,066 (72.42)	254,449 (88.66)	466,982 (92.95)
Has outpatient visits	2,963,959 (73.25)	2,893,964 (40.63)	286,214 (99.72)	230,078 (45.79)
Observation period 1 week	2,242,853 (55.63)	4,993,424 (70.11)	5254 (1.83)	223,398 (44.46)
Observation period 2 weeks	2,190,443 (54.33)	4,937,814 (69.33)	4916 (1.71)	223,203 (44.43)
Observation period 1 month	2,121,948 (52.63)	4,855,922 (68.18)	4398 (1.53)	222,915 (44.37)
Observation period 6 months	1,889,240 (46.86)	4,240,529 (59.54)	2938 (1.02)	222,006 (44.19)
Observation period 1 year	1,734,906 (43.03)	4,082,843 (57.32)	2602 (1)	221,321 (44.05)
Observation period 2 years	1,541,480 (38.23)	3,880,143 (54.48)	2241 (1)	220,300 (43.85)
Observation period 6 years	733,203 (18.18)	3,151,463 (44.25)	2007 (1)	217,257 (43.24)

a UKBB: UK Biobank.

### Cedars-Sinai

**Figure 1.**
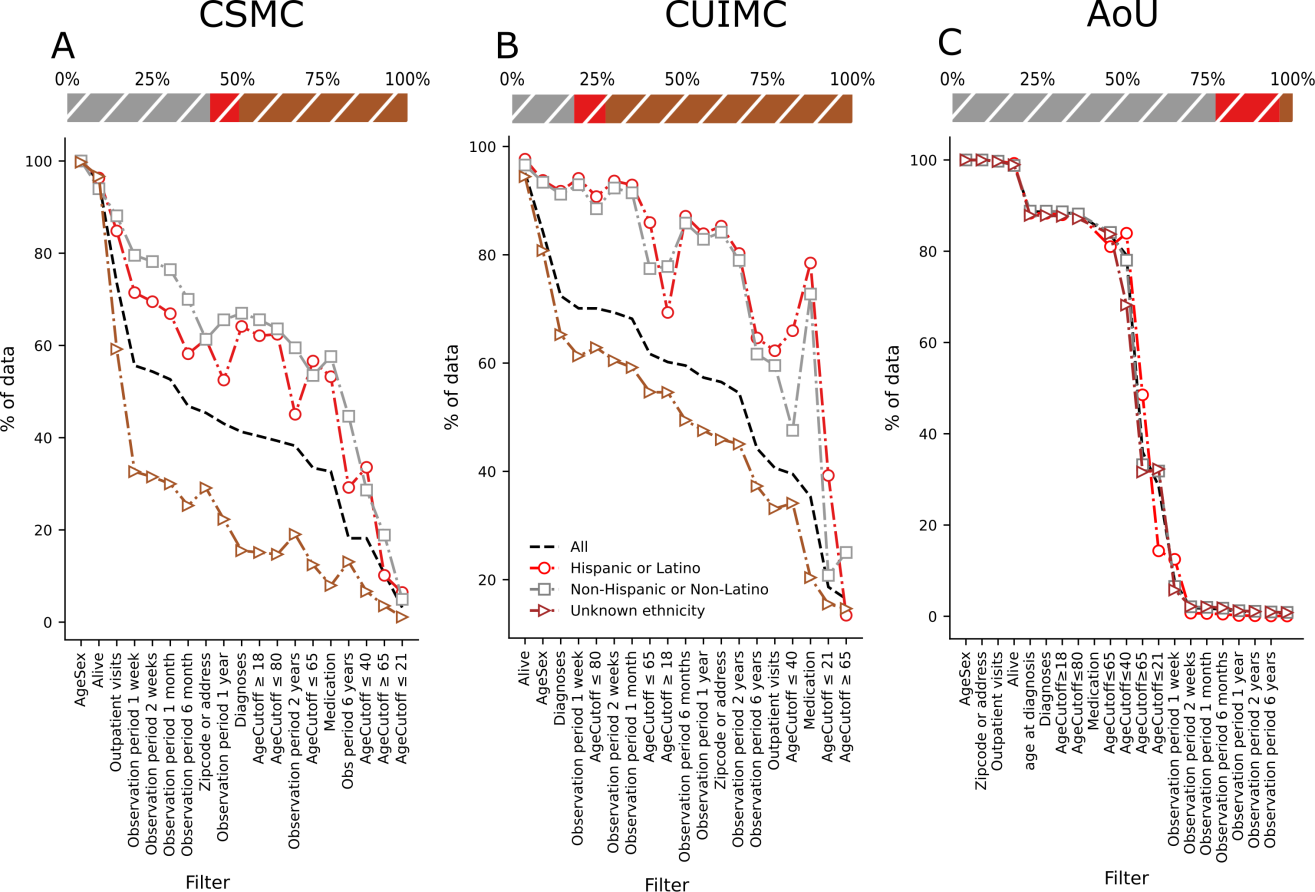
Available percentage of patients’ data upon individually applying all 19 filters in different ethnic subgroups in (A) the Cedars-Sinai dataset, (B) the CUIMC dataset, and (C) the AoU dataset. The filters are in descending order following the available percentage of the category, all. The points are connected to ease the visualization, but the filters are not cumulative. Stacked bar plots show the ethnicity distribution of the datasets in percentages. Stacked bar plot from CSMC has 8.55% (n=344,708) of Hispanic or Latino, 41.97% (n=1,691,775) of non-Hispanic or non-Latino, and 49.48% (n=1,994,824) of unknown ethnicity. Stacked bar plot from CUIMC has 9.23% (n=657,288) of Hispanic or Latino, 18.23% (n=1,298,181) of non-Hispanic or non-Latino, and 72.54% (n=5,166,379) of unknown ethnicity. Stacked bar plot from AoU has 18.83% (n=54,054) of Hispanic or Latino, 77.32% (n=221,935) of non-Hispanic or non-Latino, and 3.84% (n=11,023) of unknown ethnicity. The available percentage values can be found in Tables S2-S4 in Multimedia Appendix 1. AoU: All of Us; CSMC: Cedars-Sinai Medical Center; CUIMC: Columbia University Irving Medical Center.

Figures1A  2A show the percentage of available patients in the CSMC after applying each filter. The results show that both unknown race and unknown ethnicity are the most affected groups when applying the filters. This causes the values for the group all to decrease too, and it is shown across every cohort.

**Figure 2.**
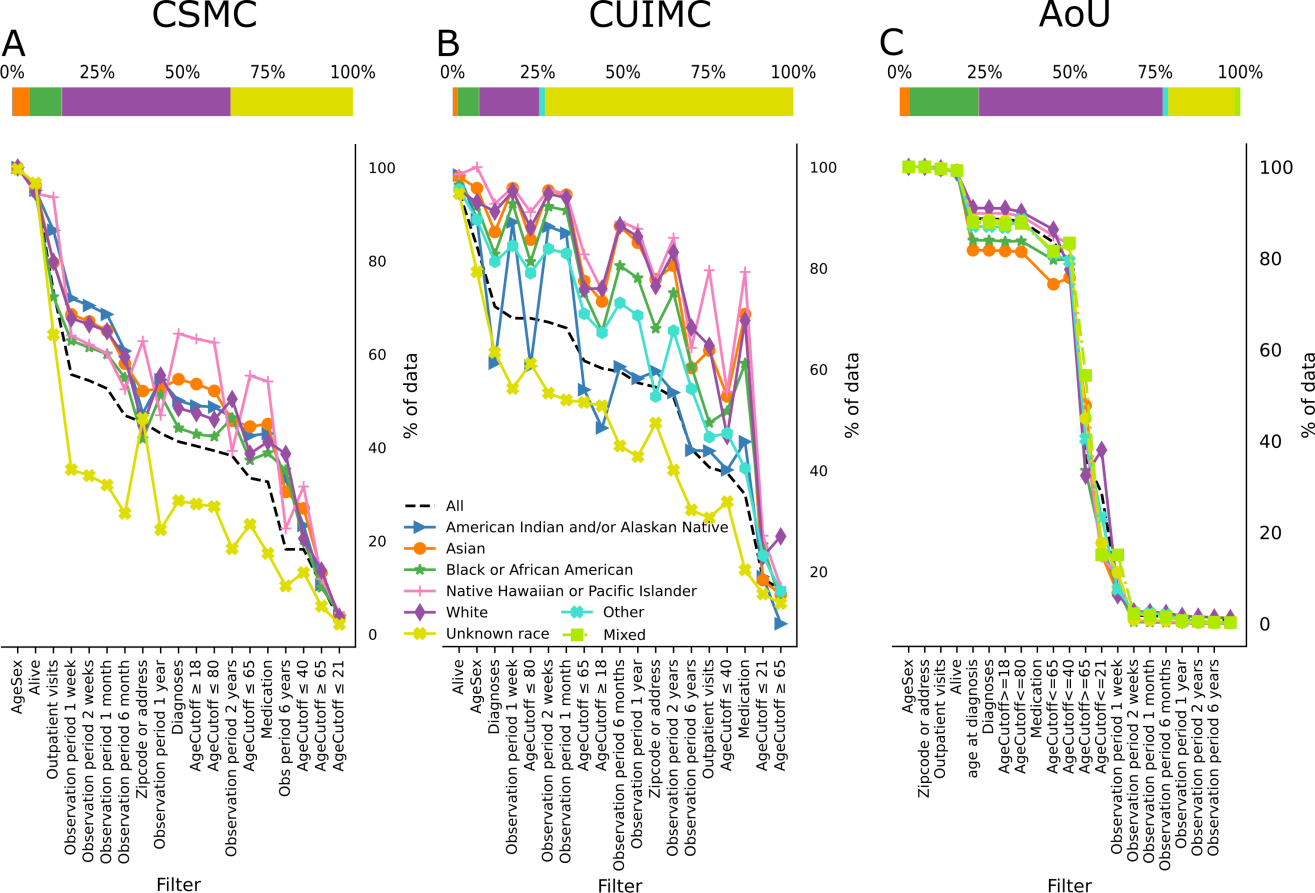
Available percentage of patients’ data upon individually applying all 19 filters in different racial subgroups in (A) the Cedars-Sinai dataset, (B) the CUIMC dataset, (C) and the AoU dataset. The filters are in descending order following the available percentage of the category, all. The points are connected to ease the visualization, but the filters are not cumulative. Stacked bar plots show the race distribution of the datasets in percentages. Stacked bar plot from CSMC has 0.14% (n=5695) of American Indian and/or Alaska Native, 5.11% (n=205,978) of Asian, 9.26% (n=373,130) of Black or African, 0.18% (n=7082) of Hawaiian Native or Pacific Islander, 49.42% (n=1,992,336) of white, and 35.89% (n=1,447,086) of unknown race. Stacked bar plot from CUIMC has 0.12% (n=8275) of American Indian and/or Alaska Native, 1.40% (n=100,046) of Asian, 6.26% (n=445,623) of Black or African, 0.09% (n=6693) of Hawaiian Native or Pacific Islander, 17.58% (n=1,252,219) of white, 14.56% (n=1,037,027) of another race, and 59.99% (n=4,271,965) of unknown race. Stacked bar plot from AoU has 2.89% (n=8294) of Asian, 20.30% (n=58,264) of Black or African, 0.12% (n=344) of Hawaiian Native or Pacific Islander, 53.89% (n=154,678) of white, 1.74% (n=5000) of mixed race, 1.65% (n=4727) of another race, and 19.40% (n=55,705) of unknown race. The available percentage values can be found in Tables S2-S4 in Multimedia Appendix 1. AoU: All of Us; CSMC: Cedars-Sinai Medical Center; CUIMC: Columbia University Irving Medical Center.

The results of the CSMC cohort show that every known race or ethnicity group is above “all” in almost every filter. However, both unknown race and unknown ethnicity are the most affected groups when applying the filters. This causes the values for the group all to decrease too, and it is shown across every cohort.

Nevertheless, it is important to note that in this dataset, the Black or African American population is the most affected group by the filters, being significantly more affected than the white population in 16 of 19 filters, as seen in Table S6 in Multimedia Appendix 1. Additionally, as the observation period filter increases, every race group becomes more affected than the white population.

### All of Us

For the AoU dataset, we applied the filters to the cohort of patients in the controlled tier 7 who had EHR records. This process reduced the number of patients from 410,235 to 287,012. Upon applying age or sex, medication, zip code or address (in this dataset, we have state of residence, so we used that instead of zip code), alive status, and outpatient visits, the initial cohort remained largely unchanged. However, as more stringent age filters were applied and the observational period was extended, the cohort population significantly decreased. Among all the races, the Asian group was most noticeably impacted, particularly when the observation period filter was applied, as shown in Figure 2A. Within this dataset, unlike at CSMC, the majority of racial and ethnic groups follow the same pattern when each filter is applied. It is remarkable that most of the groups experience significant data loss compared to the White group on the same filters, as shown in Table S7 in Multimedia Appendix 1.

### Columbia University Irving Medical Center

Similarly to the cohort from CSMC, the unknown race or ethnicity and other values decrease the most when applying the filters, bringing down the overall percentage. The known races or ethnicities are again above the all group’s percentage in almost every category. It is important to note that unknown race and ethnicity represent close to 60%(n=4,271,965) and 72%(n=5,166,379) of Columbia University Medical Center’s cohort, respectively, contributing to the low baseline percentage for the all group.

Of the known races and ethnicities, we can see in Figures1B  2B that the American Indian and/or Alaska Native population is the most significantly affected by the filters, 18 of 19, even crossing the all line. This is followed by Black or African American, which takes the second place in 16 of 19. However, contrary to the CSMC cohort, the non-Hispanic or non-Latino ethnicity is the most affected by the filters, 10 of 19 filters.

### UK Biobank

In the UKBB, race and ethnicity classifications differ from those used in American institutions. To ensure consistency in data presentation, we applied the UK government’s recommended grouping strategy [18], aligning it with the US classification system for comparability. We included any other Black background, African, Black or Black British, and Caribbean under the category Black or African origin; any other Asian background, Asian or Asian British, Bangladeshi, Chinese, Indian, and Pakistani under the category Asian; any other white background, British, Irish, and white under the category white; do not know and prefer not to answer under unknown; any other mixed background, mixed, white and Asian, white and Black African, and white and Black Caribbean under mixed; and finally, other ethnic group under other.

**Figure 3.**
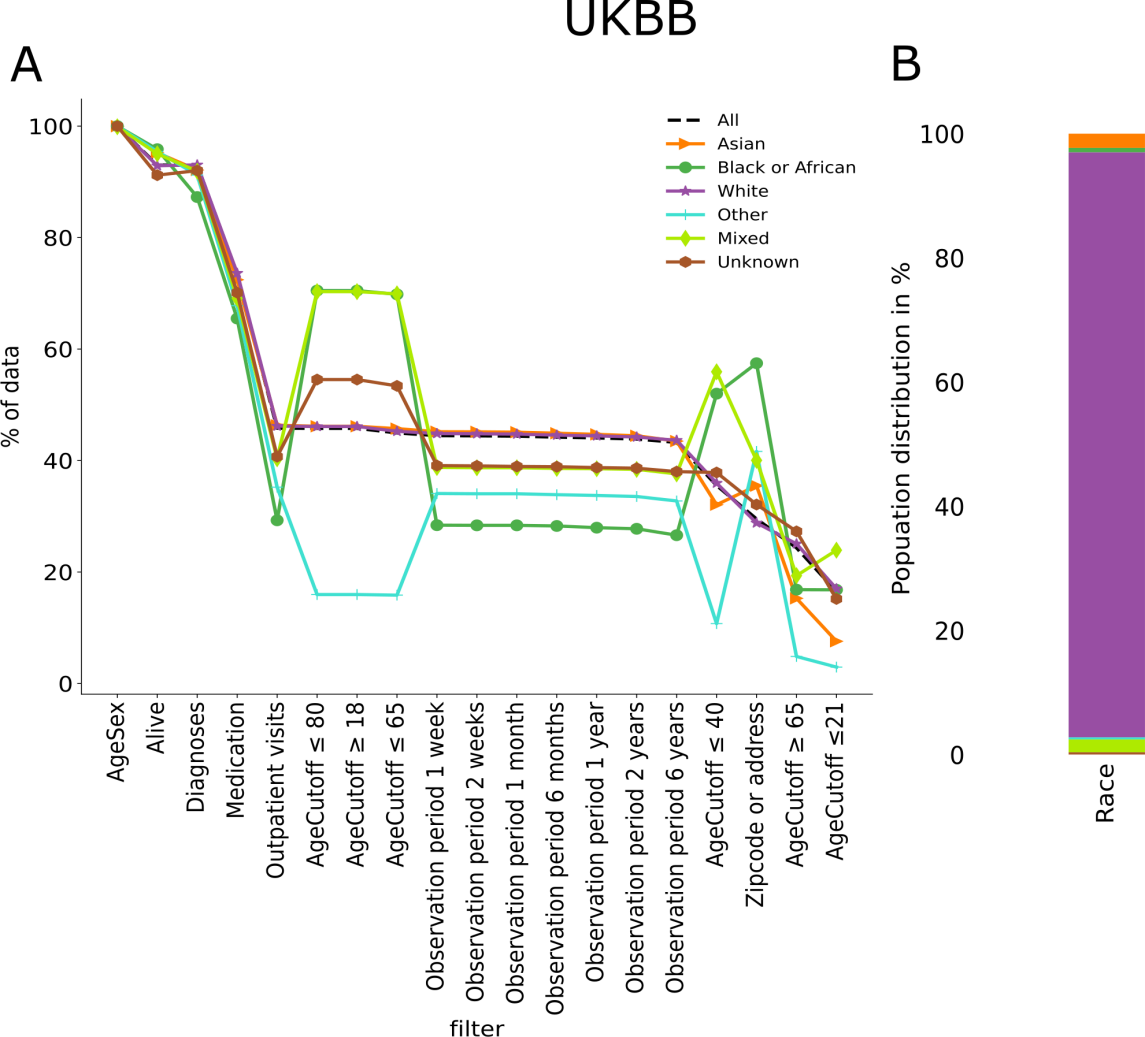
Available percentage of patients’ data upon individually applying all 19 filters in different racial subgroups (**A**) in the UKBB dataset. The filters are in descending order following the available percentage of the category, all. The points are connected to ease the visualization, but the filters are not cumulative. Stacked bar plots show the race distribution of the datasets in percentages. (B) Stacked bar plot shows the racial distribution of the dataset in percentages, showing 2.28% (n=11,472) of Asian, 0.71% (n=3552) of Black or African, 94.22% (n=473,353) of white, 0.34% (n=1731) of mixed race, 2.07% (n=10,394) of another race, and 0.38% (n=1931) of unknown race. The available percentage values can be found in Table S5 in Multimedia Appendix 1. UKBB: UK Biobank.

After this grouping, there are some aspects to remark on from this dataset, white population represents close to 94%(n=473,353) of the group, which biases completely the all results. Having that in mind, we can see in Figure 3 how every other race was impacted more than the baseline, especially the Black or African origin group.

We then analyzed the differences within the most prevalent groups in this dataset, evaluating only the 5 most common categories. We found that the British group accounts for 91%(n=442,973) of the total, only counting the top 5 groups. This approach yielded results similar to those of the complete one. The first 5 categories, in order of percentage, were the following: British (91.7%, n=442,973), ny other white background (3.4%, n=16,455), Irish (2.8%,n=13,346), Indian (1.2%, n=5,955), and other ethnic group (1%, n=4,609). These percentages account for the addition of the population of the top 5 groups and not the total.

A low adjusted *P* value (eg, <.001) suggests that the subgroup is significantly more affected by the specific filter than the white group used as a baseline. We see across every dataset that most of the groups are significantly more affected by the filter than our baseline, but it is particularly notable in groups like Black or African American and American Indian and/or Alaska Native. The Hispanic or Latino ethnic group also shows more significant data loss than non-Hispanic or non-Latino across every dataset. The *P* values can be found in Tables S6-S8 in Multimedia Appendix 1.

## Discussion

### Overview

Our study investigates the potential racial and ethnic biases introduced by applying common data quality and completeness filters in clinical research databases, including AoU, UKBB, and 2 academic medical centers. We analyzed 19 filters across approximately 12 million patients and discovered that certain filters significantly reduce data availability and have a differential effect on racial and ethnic groups.

The challenge with these filters lies in distinguishing between patients with missing data who could be relatively healthy, have not recently sought medical care, or have limited access to health care systems. Each group will have a low number of data entries in their records. Consequently, these filters might bias the resulting cohort by selecting sicker patients who interact with the health care system more frequently and/or those who have more access to health care systems. For example, in a cohort of 10,000 patients, those with poorer health status had more laboratory tests and medication orders, resulting in more comprehensive data in their records [19]. On the other hand, minoritized populations usually have less access to health care [20], which affects the data’s completeness and reduces their data points when we apply different types of filters. We focused on bringing attention to the second point.

### Principal Findings

Throughout the analysis of the 4 different cohorts, a consistent pattern emerged: applied filters disproportionately affected minoritized groups, particularly the Black or African American group, which consistently has one of the lowest data availabilities across all datasets, and the American Indian and/or Alaska Native group. These filters significantly reduced the already limited data points for minoritized groups, further diminishing the completeness and usability of their data compared to white or non-Latino patients. We observe a similar pattern in the Hispanic or Latino group, where data availability is consistently lower in every cohort compared to the non-Hispanic or non-Latino group.

In the self-reported race groups, we observe that almost every group has less data availability than the white group, which is the largest within the known self-reported races across all datasets except at CSMC. At CSMC, the most complete group varies by filter, alternating between Asian, Native Hawaiian or Pacific Islander, American Indian and/or Alaska Native, and white. In contrast, in the CUIMC dataset, the American Indian and/or Alaska Native group has the lowest data availability.

Among the 4 distinct datasets, only the AoU dataset closely reflects the diversity of the US population, with approximately 50%(n=132,334) of the data representing populations other than white. Upon applying different filters on this dataset, as shown in Figures1C  2C, most groups follow the same original pattern prior to applying the filters and deviate from the baseline to a lesser extent, demonstrating that it is possible to achieve a diverse and complete dataset.

Dataset diversity is essential for enhancing the generalizability and inclusivity of clinical research, addressing disparities, and improving health care outcomes for underrepresented populations. The AoU dataset, designed as a nationwide research program, aims to collect health data from a diverse population and succeeds at creating an equitable framework for research where most of the groups share the same data availability percentage. In contrast, the CUIMC and CSMC datasets reflect the specific patient populations of their respective regions, leading to localized diversity compared to the national scope of the AoU dataset. However, both are based in highly racially and ethnically diverse US cities, giving them a unique advantage over other institutional-level datasets. The information for the different populations and distributions of the locations for the 4 datasets can be found in Table S1 in Multimedia Appendix 1.

Both in the United States and the United Kingdom, the white population constitutes the majority. Minorities, as defined by the US Office of Management and Budget, include racial and ethnic groups such as American Indian, Alaska Native, Asian, Black or African American, and Native Hawaiian or Pacific Islander. These groups often face health disparities, which can result in reduced access to health care and underrepresentation in research cohorts [21]. This underrepresentation may lead to inaccurate clinical care decisions, skewed genetic associations, and suboptimal treatment strategies.

### Limitations

Our findings highlight the importance of carefully selecting filters to ensure equitable research outcomes, particularly for minority populations. While we do not claim these are the most frequently used filters by researchers, nor the optimal ones for selecting patients with complete data, it is essential to investigate any potential biases that may be introduced upon applying each filter before conducting research on these populations.

Additionally, methods to mitigate bias must be used when possible. One example is artificial intelligence–driven synthetic data generation for bias mitigation, which can be done using different methodologies such as generative adversarial networks, synthetic minority oversampling, or Bayesian networks [22]. Other techniques include reweighting, suppression, or multiple imputation [23]. Advanced statistical techniques like inverse probability weighting can also help address these challenges and enhance dataset diversity [24].

### Future Directions

Future work should focus on understanding how the application of these filters affects the results of common downstream analyses, such as disease risk prediction tasks and genome-wide association studies, and how to improve existing techniques for bias mitigation. We also recommend that researchers begin including sample sizes for relevant racial and ethnic groups both before and after any used filters are applied so that readers can better contextualize the results of the study.

Addressing disparities in representation is critical to creating research cohorts that accurately reflect the target population. This work underscores the challenges of achieving data completeness and proper representation of racial and ethnic populations and other minoritized groups in clinical research. Strategies to mitigate these disparities, along with careful consideration of filters, are crucial for ensuring equitable research outcomes and enhancing the inclusivity of health datasets.

### Conclusions

Our findings underscore the importance of using only necessary filters, as they may affect the diversity and completeness of sample data, which particularly affects underrepresented populations. Upon applying different filters to the 4 distinct datasets, we observed that only the AoU dataset maintained the original sample distribution along racial and ethnic groupings, with minimal deviation from the baseline, demonstrating the potential to achieve a diverse and complete dataset.

Researchers must consider their target population when conducting studies and proactively address unintentional biases that may arise in data-driven research as well as the impact of these biases on downstream analyses. While sample filters are often necessary, we recommend that researchers implement techniques to mitigate biases and provide sample size information across racial and ethnic groupings both before and after the filters are applied, so readers can better understand the generalizability of the study.

Future work should characterize how the application of these filters affects downstream analyses and improve on existing techniques to minimize their impact. We strive to achieve a state where the datasets accurately represent the target population of the studies and where research studies are performed on the same population that institutions serve.

## Supplementary material

10.2196/67591 Multimedia Appendix 1 Additional information and detailed information of the results.

## Abbreviations

AoU: All of Us
CSMC: Cedars-Sinai Medical Center
CUIMC: Columbia University Irving Medical Center
EHR: electronic health record
IRB: institutional review board
UKBB: UK Biobank
