# Supplementary material for: Biases in Race and Ethnicity Introduced by Filtering Electronic Health Records for “Complete Data”: Observational Clinical Data Analysis
Source: JMIR Med Inform. 2025 Mar 27;13:e67591. doi: 10.2196/67591 (PMC11967746; doi:10.2196/67591)
Supplement: Multimedia Appendix 1 [file medinform-v13-e67591-s001.docx]

**Table S1.** Self-reported race and ethnicity percentages of each location of the datasets, along with the total population number.

| **Dataset** | **LA County(1)** | **NY City(1)** | **US(1)** | **UK(2)** |
| --- | --- | --- | --- | --- |
| Total | 9,663,345 | 8,258,035 | 334,914,895 | 59,597,540 |
| **Race %** | | | | |
| American Indian and/or Alaska Native | 1.5 | 0.6 | 1.3 | - |
| Asian | 16.0 | 14.5 | 6.4 | 9.3 |
| Black or African | 9.0 | 23.1 | 13.7 | 4.0 |
| Native Hawaiian / Pacific Islanders | 0.4 | 0.1 | 0.3 | - |
| White | 69.6 | 37.5 | 75.3 | 81.7 |
| Mixed | 3.4 | 8.9 | 3.1 | 2.9 |
| Other | - | - | - | 2.1 |
| Unknown | - | - | - | - |
| **Ethnicity %** | | | | |
| Hispanic or Latino | 48.6 | 29.0 | 19.5 | - |
| Non-Hispanic or Non-Latino | 51.4 | 71.0 | 80.5 | - |
| Unknown | - | - | - | - |

**Table S2.** Available percentage of patients' data upon applying all the 19 filters to the Cedars-Sinai Medical Center dataset. The underlined and bold text indicates the most affected known race by that filter, and the bold text indicates the most affected known ethnicity.

| Filter | All | American I. and/or Alaskan N. | Asian | Black or African American | Native Hawaiian or Pacific Islander | White | Unknown race | Hispanic or Latino | Non- Hispanic or non-Latino | Unknown ethnicity |
| --- | --- | --- | --- | --- | --- | --- | --- | --- | --- | --- |
| Alive | 95.56 | 95.14 | 96.01 | 94.60 | **94.41** | 94.78 | 96.67 | 96.31 | **93.98** | 96.66 |
| AgeSex | 99.87 | 100.00 | 100.00 | 100.00 | 100.00 | 100.00 | 99.64 | 100.00 | 100.00 | 99.74 |
| AgeCutoff ≥ 18 | 40.31 | 48.90 | 53.60 | **42.86** | 63.27 | 47.36 | 27.90 | **62.11** | 65.58 | 15.11 |
| AgeCutoff ≤ 21 | 3.17 | 04.07 | **3.54** | 4.33 | 4.56 | 3.65 | 2.14 | 6.44 | **4.94** | 1.11 |
| AgeCutoff ≤ 40 | 18.19 | 23.30 | 26.96 | **20.19** | 31.64 | 20.47 | 13.20 | 33.57 | **28.65** | 6.66 |
| AgeCutoff ≤ 65 | 33.44 | 42.53 | 44.49 | **37.29** | 55.41 | 38.68 | 23.51 | 56.62 | **53.49** | 12.43 |
| AgeCutoff ≥ 65 | 10.54 | 10.64 | 13.18 | **9.98** | 11.82 | 13.64 | 06.02 | **10.15** | 18.88 | 3.53 |
| AgeCutoff ≤ 80 | 39.31 | 48.69 | 52.14 | **42.36** | 62.48 | 45.99 | 27.33 | **62.42** | 63.60 | 14.71 |
| Zip or address | 45.38 | 47.32 | 52.09 | **41.87** | 62.79 | 44.62 | 46.27 | 61.35 | **61.33** | 29.09 |
| Medication | 32.66 | 43.00 | 45.02 | **38.84** | 54.17 | 41.26 | 17.32 | **53.22** | 57.60 | 7.96 |
| Diagnoses | 41.26 | 50.04 | 54.62 | **44.17** | 64.40 | 48.43 | 28.60 | **64.12** | 66.99 | 15.49 |
| Outpatient visits | 73.52 | 86.55 | 79.62 | **72.28** | 93.65 | 79.80 | 64.18 | **84.83** | 88.11 | 59.20 |
| Obs period 1 w | 55.64 | 71.98 | 68.52 | **62.86** | 63.98 | 67.66 | 35.27 | **71.45** | 79.54 | 32.63 |
| Obs period 2 w | 54.34 | 70.43 | 67.03 | **61.47** | 62.07 | 66.40 | 33.98 | **69.49** | 78.19 | 31.49 |
| Obs period 1 m | 52.64 | 68.53 | 65.02 | **60.00** | 60.15 | 64.92 | 31.97 | **66.89** | 76.45 | 29.98 |
| Obs period 6 m | 46.86 | 60.68 | 57.98 | 54.96 | **52.46** | 59.36 | 25.91 | **58.19** | 70.00 | 25.29 |
| Obs period 1 y | 43.04 | 54.52 | 52.69 | 51.29 | **46.92** | 55.43 | 22.40 | **52.49** | 65.56 | 22.30 |
| Obs period 2 y | 38.24 | 46.39 | 45.73 | 46.32 | **39.27** | 50.39 | 18.32 | **45.07** | 59.50 | 19.03 |
| Obs period 6 y | 18.19 | 33.22 | 30.51 | 35.16 | **22.66** | 38.63 | 10.34 | **29.21** | 44.64 | 13.08 |

**Table S3.** Available percentage of patients' data upon applying all the 19 filters to the Columbia University Irving Medical Center dataset. The underlined and bold text indicates the most affected known race by that filter, and the bold text indicates the most affected known ethnicity.

| Filter | All | American I. and/or Alaska N. | Asian | Black or African American | Native Hawaiian or Pacific Islander | White | Other race | Unknown ethnicity | Hispanic or Latino | Non- Hispanic or non-Latino | Unknown race |
| --- | --- | --- | --- | --- | --- | --- | --- | --- | --- | --- | --- |
| Alive | 95.13 | 98.42 | 98.06 | 96.43 | 98.55 | **95.26** | 95.61 | 94.44 | 97.66 | **96.58** | 94.75 |
| AgeSex | 84.28 | **89.56** | 95.83 | 92.26 | 100.00 | 93.08 | 89.58 | 80.79 | 93.74 | **93.40** | 79.28 |
| AgeCutoff ≥18 | 60.19 | **48.41** | 73.42 | 67.41 | 75.54 | 76.01 | 67.24 | 54.60 | **69.35** | 77.84 | 52.78 |
| AgeCutoff ≤ 21 | 18.65 | 19.01 | **18.3**2 | 25.29 | 27.10 | 23.00 | 23.17 | 15.50 | 39.24 | **20.79** | 15.58 |
| AgeCutoff ≤ 40 | 39.52 | **40.12** | 54.66 | 51.75 | 55.58 | 46.81 | 47.39 | 34.12 | 65.98 | **47.58** | 33.81 |
| AgeCutoff ≤ 65 | 61.69 | **55.98** | 77.46 | 75.12 | 82.74 | 75.91 | 70.98 | 54.64 | 85.95 | **77.45** | 53.47 |
| AgeCutoff ≥ 65 | 16.43 | **9.69** | 15.44 | 13.92 | 16.96 | 26.98 | 16.13 | 14.66 | **13.43** | 25.02 | 13.71 |
| AgeCutoff ≤ 80 | 70.10 | **60.65** | 85.66 | 81.29 | 91.10 | 88.04 | 79.00 | 62.85 | 90.75 | **88.51** | 61.14 |
| Zip or address | 56.50 | **59.63** | 77.81 | 68.06 | 77.63 | 76.48 | 54.64 | 45.89 | 85.29 | **84.17** | 49.36 |
| Medication | 35.30 | **45.69** | 70.91 | 61.18 | 79.26 | 69.66 | 40.43 | 20.39 | 78.49 | **72.75** | 20.35 |
| Diagnoses | 72.43 | **61.23** | 87.16 | 82.78 | 92.75 | 91.20 | 81.27 | 65.25 | 91.75 | **91.19** | 63.34 |
| Outpatient visits | 40.64 | **43.87** | 63.74 | 49.42 | 79.59 | 64.66 | 46.59 | 33.13 | 62.28 | **59.55** | 30.62 |
| Obs period 1 w | 70.11 | **89.12** | 95.81 | 92.75 | 96.01 | 95.22 | 84.44 | 61.32 | 94.12 | **92.95** | 56.24 |
| Obs period 2 w | 69.33 | **88.25** | 95.36 | 92.18 | 95.49 | 94.69 | 83.85 | 60.46 | 93.63 | **92.33** | 55.31 |
| Obs period 1 m | 68.18 | **86.92** | 94.53 | 91.43 | 94.71 | 93.94 | 82.93 | 59.19 | 92.90 | **91.45** | 53.93 |
| Obs period 6 m | 59.54 | **60.52** | 88.29 | 80.49 | 89.23 | 88.44 | 73.15 | 49.42 | 87.12 | **85.85** | 44.86 |
| Obs period 1 y | 57.33 | **58.19** | 85.04 | 78.05 | 87.79 | 86.23 | 70.65 | 47.53 | 83.90 | **82.86** | 42.76 |
| Obs period 2 y | 54.48 | **55.42** | 80.49 | 75.13 | 86.00 | 83.15 | 67.64 | 45.05 | 80.24 | **78.96** | 40.07 |
| Obs period 6 y | 44.25 | **43.99** | 60.27 | 60.61 | 64.25 | 68.27 | 56.15 | 37.28 | 64.64 | **61.66** | 32.21 |

**Table S4.** Available percentage of patients' data upon applying all the 19 filters to the All of Us dataset. The underlined and bolded text indicates the most affected known race by that filter, and the bold text indicates the most affected known ethnicity.

| Filter | All | Asian | Black or African American | Mixed | Native Hawaiiand/or Pacific Islander | White | Other | Unknown race | Hispanic or Latino | Non- Hispanic or non-Latino | Unknown ethnicity |
| --- | --- | --- | --- | --- | --- | --- | --- | --- | --- | --- | --- |
| Alive | 98.88 | 99.28 | 98.75 | 99.20 | **98.55** | 98.78 | 98.98 | 99.21 | **99.25** | 98.79 | 98.95 |
| AgeSex | 100.00 | 100.00 | 100.00 | 100.00 | 100.00 | 100.00 | 100.00 | 100.00 | 100.00 | 100.00 | 100.00 |
| AgeCutoff ≥ 18 | 88.49 | **81.59** | 83.70 | 87.40 | 89.83 | 90.89 | 86.72 | 88.13 | **88.00** | 88.65 | 87.66 |
| AgeCutoff ≤ 21 | 7.62 | 6.62 | 7.50 | 15.04 | 10.47 | **6.19** | 7.49 | 11.21 | 12.48 | **6.53** | 5.76 |
| AgeCutoff ≤ 40 | 36.04 | 47.75 | 33.53 | 54.34 | 40.99 | **32.42** | 40.45 | 44.92 | 48.53 | **32.21** | 31.62 |
| AgeCutoff ≤ 65 | 79.12 | **75.74** | 79.76 | 83.34 | 83.14 | 77.54 | 79.35 | 82.91 | 83.94 | **78.03** | 68.20 |
| AgeCutoff ≥ 65 | 28.55 | 15.11 | 17.41 | 15.04 | **14.53** | 38.01 | 23.27 | 17.67 | **14.34** | 31.83 | 32.27 |
| AgeCutoff ≤ 80 | 88.14 | **81.41** | 83.77 | 87.62 | 89.24 | 90.17 | 88.51 | 88.09 | **88.07** | 88.16 | 87.15 |
| Zip or address | 100.00 | 100.00 | 100.00 | 100.00 | 100.00 | 100.00 | 100.00 | 100.00 | 100.00 | 100.00 | 100.00 |
| Medication | 83.51 | **74.31** | 79.63 | 81.52 | 84.88 | 86.27 | 81.00 | 81.66 | **81.00** | 84.11 | 83.73 |
| Diagnoses | 88.65 | **81.75** | 83.92 | 87.82 | 89.83 | 90.97 | 86.93 | 88.40 | **88.31** | 88.78 | 87.83 |
| Outpatient visits | 99.72 | 99.35 | **99.86** | 99.56 | 100.00 | 99.70 | 99.56 | 99.72 | **99.72** | 99.73 | 99.66 |
| Obs period 1 w | 1.83 | 2.63 | **0.35** | 2.16 | 0.58 | 2.67 | 2.41 | 0.85 | **0.69** | 2.09 | 2.17 |
| Obs period 2 w | 1.71 | 2.41 | **0.29** | 1.82 | 0.58 | 2.53 | 2.28 | 0.77 | **0.61** | 1.96 | 2.08 |
| Obs period 1 m | 1.53 | 1.97 | **0.24** | 1.48 | 0.58 | 2.32 | 1.97 | 0.62 | **0.47** | 1.78 | 1.86 |
| Obs period 6 m | 1.02 | 0.76 | 0.09 | 0.66 | **0.00** | 1.67 | 0.87 | 0.31 | **0.17** | 1.22 | 1.21 |
| Obs period 1 y | 0.91 | 0.49 | 0.06 | 0.48 | **0.00** | 1.51 | 0.59 | 0.24 | **0.12** | 1.09 | 1.03 |
| Obs period 2 y | 0.78 | 0.30 | 0.02 | 0.32 | **0.00** | 1.34 | 0.28 | 0.19 | **0.07** | 0.95 | 0.93 |
| Obs period 6 y | 0.70 | 0.17 | 0.01 | 0.24 | **0.00** | 1.21 | 0.19 | 0.17 | **0.06** | 0.85 | 0.81 |

**Table S5.** Available percentage of patients' data upon applying all the 19 filters to the UK Biobank dataset. The underlined and bolded text indicates the most affected known race by that filter.

| Filter | All | Asian | Black or African | Mixed | White | Other | Unknown |
| --- | --- | --- | --- | --- | --- | --- | --- |
| Alive | 93.03 | 95.23 | 95.86 | 95.03 | **92.89** | 95.44 | 91.20 |
| AgeSex | 100.00 | 99.99 | 100.00 | **99.88** | 99.98 | 99.99 | 100.00 |
| AgeCutoff ≥ 18 | 45.78 | 46.15 | 70.50 | 70.31 | **46.10** | 15.96 | 54.53 |
| AgeCutoff ≤ 21 | 16.51 | **7.57** | 16.81 | 23.92 | 17.00 | 2.94 | 15.17 |
| AgeCutoff ≤ 40 | 35.53 | **32.08** | 52.00 | 55.92 | 35.95 | 10.75 | 37.86 |
| AgeCutoff ≤ 65 | 44.95 | 45.73 | 69.82 | 69.90 | **45.25** | 15.86 | 53.39 |
| AgeCutoff ≥ 65 | 24.35 | **15.28** | 16.84 | 19.35 | 25.06 | 4.85 | 27.29 |
| AgeCutoff ≤ 80 | 45.78 | 46.15 | 70.50 | 70.31 | **46.10** | 15.96 | 54.53 |
| Zip or address | 29.51 | 35.49 | 57.46 | 40.03 | **28.80** | 41.64 | 32.11 |
| Medication | 73.37 | 72.44 | **65.48** | 69.09 | 73.61 | 67.78 | 70.12 |
| Diagnoses | 92.96 | 92.13 | **87.27** | 91.91 | 93.05 | 91.22 | 92.02 |
| Outpatient visits | 45.80 | 46.34 | **29.28** | 40.32 | 46.18 | 35.22 | 40.70 |
| Obs period 1 w | 44.47 | 45.17 | **28.41** | 38.76 | 44.84 | 34.09 | 39.10 |
| Obs period 2 w | 44.43 | 45.15 | **28.38** | 38.71 | 44.80 | 34.04 | 39.05 |
| Obs period 1 m | 44.37 | 45.09 | **28.38** | 38.71 | 44.74 | 34.04 | 38.94 |
| Obs period 6 m | 44.19 | 44.90 | **28.27** | 38.59 | 44.56 | 33.88 | 38.89 |
| Obs period 1 y | 44.06 | 44.70 | **27.96** | 38.53 | 44.42 | 33.74 | 38.74 |
| Obs period 2 y | 43.85 | 44.45 | **27.76** | 38.42 | 44.22 | 33.55 | 38.63 |
| Obs period 6 y | 43.25 | 43.49 | **26.60** | 37.61 | 43.63 | 32.75 | 38.01 |

**Table S6.** P-Values of binomial tests from Cedars Sinai dataset. After applying the Bonferroni correction, significant P values are those under 0.0003, in the table shown as <.001. A significant P value indicates that the group (column) is more affected by the filter (row) than the group white.

| **Filters** | **American Indian and/or Alaska N.** | **Asian** | **Black or African American** | **Hispanic or Latino** | **Native Hawaiian and/or Pacific I.** | **Non- Hispanic or Latino** | **Unknown** | **Unknown race** |
| --- | --- | --- | --- | --- | --- | --- | --- | --- |
| AgeSex P value | .50 | .50 | .50 | .50 | .50 | .50 | <.001 | .004 |
| AgeCutoff ≥ 18 P value | .95 | 1 | <.001 | 1 | 1 | 1 | <.001 | <.001 |
| AgeCutoff ≤ 21 P value | .95 | .006 | 1 | 1 | 1 | 1 | <.001 | <.001 |
| AgeCutoff ≤ 40 P value | 1 | 1 | <.001 | 1 | 1 | 1 | <.001 | <.001 |
| AgeCutoff ≤ 65 P value | 1 | 1 | <.001 | 1 | 1 | 1 | <.001 | <.001 |
| AgeCutoff ≥ 65 P value | <.001 | <.001 | <.001 | <.001 | <.001 | 1 | <.001 | <.001 |
| AgeCutoff ≤ 80 P value | .99 | 1 | <.001 | 1 | 1 | 1 | <.001 | <.001 |
| Alive P value | .61 | 1 | .14 | 1 | .37 | <.001 | 1 | 1 |
| Diagnoses P value | .96 | 1 | <.001 | 1 | 1 | 1 | <.001 | <.001 |
| Medication P value | .97 | 1 | <.001 | 1 | 1 | 1 | <.001 | <.001 |
| Outpatient visits P value | 1 | .19 | <.001 | 1 | 1 | 1 | <.001 | <.001 |
| Zip or address P value | .99 | 1 | <.001 | 1 | 1 | 1 | 1 | <.001 |
| Obs period 1 week P value | 1 | 1 | <.001 | 1 | <.001 | 1 | <.001 | <.001 |
| Obs period 2 weeks P value | 1 | 1 | <.001 | 1 | <.001 | 1 | <.001 | <.001 |
| Obs period 1 month P value | 1 | .71 | <.001 | 1 | <.001 | 1 | <.001 | <.001 |
| Obs period 6 month P value | .90 | <.001 | <.001 | <.001 | <.001 | 1 | <.001 | <.001 |
| Obs period 1 year P value | .18 | <.001 | <.001 | <.001 | <.001 | 1 | <.001 | <.001 |
| Obs period 2 years P value | <.001 | <.001 | <.001 | <.001 | <.001 | 1 | <.001 | <.001 |
| Obs period 6 years P value | <.001 | <.001 | <.001 | <.001 | <.001 | 1 | <.001 | <.001 |

**Table S7.** P-Values of binomial tests from All of Us dataset. After applying the Bonferroni correction, significant P values are those under 0.0003, in the table shown as <.001. A significant P value indicates that the group (column) is more affected by the filter (row) than the group white.

| **Filter** | **Asian** | **Black or African American** | **Native Hawaiian and/or Pacific Islander** | **Mixed** | **Other** | **Non-Hispanic or non-Latino** | **Hispanic or Latino** | **Unknown ethnicity** | **Unknown race** |
| --- | --- | --- | --- | --- | --- | --- | --- | --- | --- |
| AgeSex P value | 0.50 | 0.50 | 0.51 | 0.50 | 0.50 | 0.50 | 0.50 | 0.50 | 0.50 |
| AgeCutoff ≥ 18 P value | <0.001 | <0.001 | 0.43 | 0.01 | 0.002 | <0.001 | <0.001 | <0.001 | <0.001 |
| AgeCutoff ≥ 65 P value | <0.001 | <0.001 | <0.001 | <0.001 | <0.001 | <0.001 | <0.001 | <0.001 | <0.001 |
| AgeCutoff=<21 P value | 0.94 | 1 | 1 | 1 | 1 | 1 | 1 | 0.04 | 1 |
| AgeCutoff=<40 P value | 1 | 1 | 1 | 1 | 1 | 1 | 1 | 0.08 | 1 |
| AgeCutoff=<65 P value | 0.04 | 1 | 0.89 | 1 | 0.92 | 0.95 | 1 | <0.001 | 1 |
| AgeCutoff=<80 P value | <0.001 | <0.001 | 0.44 | 0.03 | 0.12 | <0.001 | <0.001 | <0.001 | <0.001 |
| Alive P value | 0.67 | 0.48 | 0.50 | 0.62 | 0.56 | 0.51 | 0.83 | 0.57 | 0.81 |
| Obs period 1 week P value | 0.42 | <0.001 | 0.005 | 0.01 | 0.15 | <0.001 | <0.001 | 0.001 | <0.001 |
| Obs period 2 weeks P value | 0.26 | <0.001 | 0.008 | 0.001 | 0.157 | <0.001 | <0.001 | 0.002 | <0.001 |
| Obs period 1 month P value | 0.02 | <0.001 | 0.01 | <0.001 | <0.001 | <0.001 | <0.001 | 0.001 | <0.001 |
| Obs period 6 months P value | <0.001 | <0.001 | 0.003 | <0.001 | <0.001 | <0.001 | <0.001 | <0.001 | <0.001 |
| Obs period 1 year P value | <0.001 | <0.001 | 0.005 | <0.001 | <0.001 | <0.001 | <0.001 | <0.001 | <0.001 |
| Obs period 2 years P value | <0.001 | <0.001 | 0.01 | <0.001 | <0.001 | <0.001 | <0.001 | <0.001 | <0.001 |
| Obs period 6 years P value | <0.001 | <0.001 | 0.01 | <0.001 | <0.001 | <0.001 | <0.001 | <0.001 | <0.001 |
| Diagnoses P value | <0.001 | <0.001 | 0.43 | 0.01 | <0.001 | <0.001 | <0.001 | <0.001 | <0.001 |
| Medication P value | <0.001 | <0.001 | 0.41 | <0.001 | <0.001 | <0.001 | <0.001 | 0.003 | <0.001 |
| Outpatient visits P value | 0.38 | 0.63 | 0.54 | 0.46 | 0.46 | 0.52 | 0.51 | 0.48 | 0.51 |
| Zipcode or address P value | 0.50 | 0.50 | 0.52 | 0.51 | 0.51 | 0.50 | 0.50 | 0.50 | 0.50 |

**Table S8.** P Values of binomial tests from Columbia University Irving Medical Center dataset. After applying the Bonferroni correction, significant P values are those under 0.0003, in the table shown as <.001. A significant P value indicates that the group (column) is more affected by the filter (row) than the group white.

| **Filters** | **American I. and/or Alaska N.** | **Asian** | **Black or African American** | **Hispanic or Latino** | **Native Hawaiian and/or Pacific I.** | **Non-Hispanic or non-Latino** | **Other** | **Unknown ethnicity** | **Unknown race** |
| --- | --- | --- | --- | --- | --- | --- | --- | --- | --- |
| AGE<=65 P value | <.001 | 1 | <.001 | 1 | 1 | 1 | <.001 | <.001 | <.001 |
| AGE<=80 P value | <.001 | <.001 | <.001 | 1 | .99 | 1 | <.001 | <.001 | <.001 |
| Age<=40 P value | <.001 | 1 | 1 | 1 | 1 | 1 | 1 | <.001 | <.001 |
| Age<=21 P value | <.001 | <.001 | 1 | 1 | 1 | <.001 | .99 | <.001 | <.001 |
| Age ≥ 18 P value | <.001 | <.001 | <.001 | <.001 | .33 | 1 | <.001 | <.001 | <.001 |
| Age ≥ 65 P value | <.001 | <.001 | <.001 | <.001 | <.001 | <.001 | <.001 | <.001 | <.001 |
| AgeSex P value | <.001 | 1 | <.001 | 1 | 1 | .99 | <.001 | <.001 | <.001 |
| Alive P value | .99 | 1 | 1 | 1 | .99 | 1 | .99 | <.001 | <.001 |
| Diagnoses P value | <.001 | <.001 | <.001 | 1 | .90 | .46 | <.001 | <.001 | <.001 |
| Obs period 14 days P value | <.001 | .98 | <.001 | <.001 | .75 | <.001 | <.001 | <.001 | <.001 |
| Obs period 180 days P value | <.001 | .31 | <.001 | <.001 | .75 | <.001 | <.001 | <.001 | <.001 |
| Obs period 2 years P value | <.001 | <.001 | <.001 | <.001 | .99 | <.001 | <.001 | <.001 | <.001 |
| Obs period 30 days P value | <.001 | .96 | <.001 | <.001 | .74 | <.001 | <.001 | <.001 | <.001 |
| Obs period 365 days P value | <.001 | <.001 | <.001 | <.001 | .91 | <.001 | <.001 | <.001 | <.001 |
| Obs period 6 years P value | <.001 | <.001 | <.001 | <.001 | <.001 | <.001 | <.001 | <.001 | <.001 |
| Obs period 7 days P value | <.001 | .96 | <.001 | <.001 | .74 | <.001 | <.001 | <.001 | <.001 |
| Outpatient P value | <.001 | <.001 | <.001 | <.001 | 1 | <.001 | <.001 | <.001 | <.001 |
| Address P value | <.001 | 1 | <.001 | 1 | .86 | 1 | <.001 | <.001 | <.001 |
| Medications P value | <.001 | 1 | <.001 | 1 | 1 | 1 | <.001 | <.001 | <.001 |


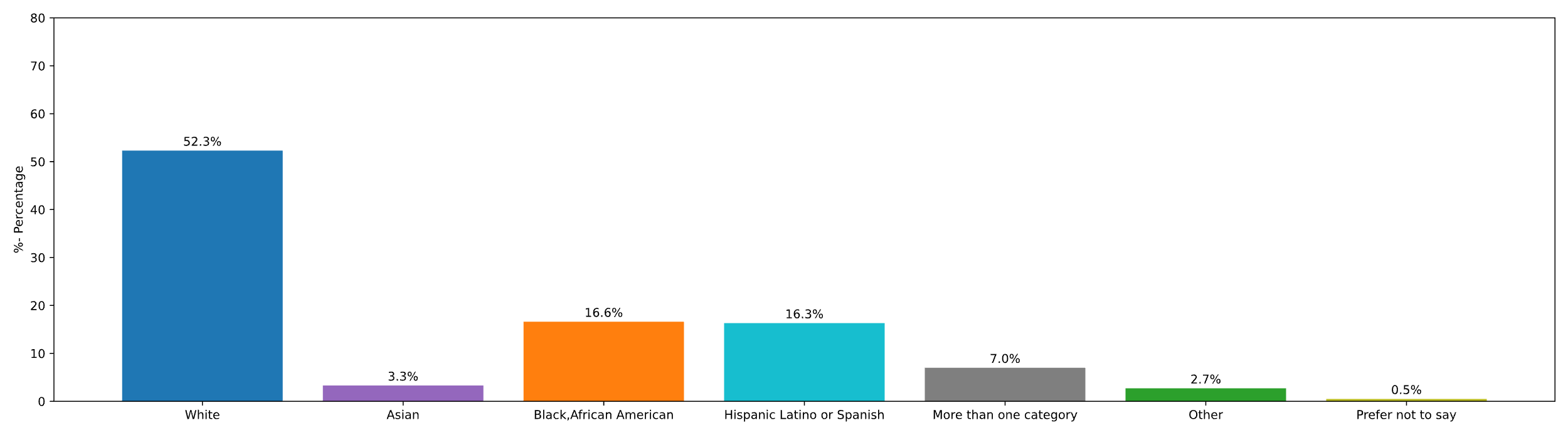


**Figure S1.** Distribution of the self-reported races and ethnicities of the participants who have completed the initial steps of the program, with or without EHR data(3).

**References**

1. www.census.gov [Internet]. United States Census Bureau. Available from: https://www.census.gov/quickfacts/fact/table/newyorkcitynewyork,losangelescountycalifornia,US/PST045223

2. gov.uk [Internet]. Population of England and Wales. Available from: https://www.ethnicity-facts-figures.service.gov.uk/uk-population-by-ethnicity/national-and-regional-populations/population-of-england-and-wales/latest/

3. All of Us Research Program. Researcher Workbench [Internet]. 2023. Available from: https://www.researchallofus.org/data-tools/workbench/
